# Donors' Quality of Life after Living Donor Liver Transplantation: Shiraz Organ Transplant Center Experience

**Published:** 2020

**Authors:** A. Shamsaeefar, S. Nikeghbalian, K. Kazemi, S. Gholami, M. Sayadi, F. Azadian, N. Motazedian, S. A. Malek-Hosseini

**Affiliations:** 1 *Shiraz Organ Transplant Center, Shiraz University of Medical Sciences, Shiraz, Iran*; 2 *Cardiovascular Research Center, Shiraz University of Medical Sciences, Shiraz, Iran*; 3 *Non-Communicable Disease Research Center, Shiraz University of Medical Sciences, Shiraz, Iran*; 4 *Shiraz Transplant Research Center, Shiraz University of Medical Sciences, Shiraz, Iran*

**Keywords:** Liver transplantation, Quality of life, Donor, Living donor

## Abstract

**Background:**

Probable effects of living donor liver transplantation on the wellbeing of the donor and psychological difficulties are necessary to be understood.

**Objective:**

To assess the quality of life of living donors after liver donation.

**Methods:**

140 living donors who underwent hepatectomy between 2012 and July 2015 were enrolled in this study. Donors were asked to complete the Short Form 36-question Health Survey (SF-36) through face to face or by telephone interview.

**Results:**

The mean±SD age of donors at transplantation was 32.1±7.3 years; 83 (59.3%) of donors were female. 134 (95.7%) were married. The mean±SD BMI was 23.8±3.5 (kg/m^2^). “Mother-to-child” was the most frequent relationship (n=79, 56.4%). 22 (15.7%) complications were reported by participants. The mean±SD score of Physical Component Summary and Mental Component Summary were 48.8±14.6 and 50.1±6.9, respectively.

**Conclusion:**

Most living donors sustain a near average quality of life post-donation. It seems that living donation does not negatively affect the quality of life.

## INTRODUCTION

New surgical methods have been developed in the field of liver transplantation to overcome organ shortage such as split liver organ, living donor, and reduced size [1]. Living donor liver transplantation (LDLT) is a volunteer action in which a healthy person denotes a fragment of his or her healthy liver to a liver recipient [2, 3]. A review article in 2015 reported a morbidity rate of 8.6% to 59% and a mortality rate of 0.2% among LDLT donors. The most common complication for donors was biliary complication and biliary leak [4]. The most commonly reported complications among living donors were in Clavien grades 1 and 2 [5, 6].

A study from China showed temporary abnormalities in liver function test and blood count among many of 300 living donors. While laboratory tests could be used to identify some post-operation complications, they are not useful to detect some physical, mental, and psychological difficulties, which mostly influence the quality of life of the donors after transplantation [7]. Nor can we evaluate the donor physical and psychological health solely based on common measured surgical factors[5]. Health-related quality of life is assessed in various ways, and influenced by several factors. One way to measure the quality of life is using questionnaires such as the Short Form (SF)-36 Health Survey [8, 9].

One study showed the Physical Component Score (PCS) decreased immediately after donation, then returned to the baseline within 6–12 months, while the Mental Component Score (MCS) remains comparable to that of normative population throughout the procedure [4, 10].

Living donors may experience various complications that are usually mild and have a good prognosis [11]. The probable effect of LDLT on the wellbeing of the donor and the psychological difficulties they might experience should be understood. We, therefore, conducted this study to assess the quality of life of living donors post liver-donation surgery.

## METHODS AND MATERIALS

We retrospectively reviewed data of donors who underwent hepatectomy for liver transplantation at Shiraz Organ Transplant Center from 2012 to 2015. In census way, a total of 140 living donors underwent hepatectomy during this period. In the course of the donor evaluation process, all patients gave their informed written consent to participate in the follow-up studies. Our study was approved by the Ethical Committee of Shiraz University of Medical Sciences. The donors were asked to complete a data collecting form and the SF-36 through face to face or by telephone interview. The self-administered SF-36 survey assesses eight health domains: physical functioning (PF), role physical (RP), bodily pain (BP), general health (GH), vitality (VT), social functioning (SF), role emotional (RE), and mental health (MH). Component analyses showed that there are two distinct concepts measured by the SF-36: a physical dimension, represented by the Physical Component Summary (PCS); and a mental dimension, represented by the Mental Component Summary (MCS). The eight subscales are summarized by PCS and the MCS. The SF-36 score for each question ranged from 0 to 100 with higher score representative of better function. The general population average is 50 with a standard deviation of 10 [12]. We classified post-operative complications among liver donors according to the Clavien system (Table 1).

**Table 1:** Classification of complications according to the Clavien system

Grade	Complication
1	Any deviation from the normal post-operative course without the need for pharmacological treatment or surgical, endoscopic, and radiological interventions. Allowed therapeutic regimens are drugs as antiemetics, antipyretics, analgesics, diuretics, electrolytes, and physiotherapy. This grade also includes wound infections opened at the bedside.
2	Complications requiring pharmacological treatment with drugs other than such allowed for grade 1 complications. Blood transfusions and total parenteral nutrition are also included.
3	Complications requiring surgical, endoscopic, or radiological intervention
3a	Intervention not under general anesthesia
3b	Intervention under general anesthesia
4	Life-threatening complications (including central nervous system complications) requiring intensive care unit stay
4a	Single organ dysfunction (including dialysis)
4b	Multiorgan dysfunction
5	Death of the patient

Most of the additional data used for analysis were obtained by chart review, anesthesia records, and the computerized hospital database. Continues variables were expressed as mean±SD; categorical variables, number (percent). Student’s t test for independent samples, one-way ANOVA, and Person’s correlation coefficient were used for data analysis. A p value <0.05 was considered statistically significant.

## Results

Donor Characteristics

The mean±SD age of donors at transplantation time was 32.1±7.3 (range: 17–65) years; 83 (59.3%) of them were female. Most of the donors (n=134, 95.7%) were married. About one-third (n=45, 32.2%) of donors had a diploma and higher education. Among our participants, 61 (43.6%) were employed. The mean±SD for Body Mass Index (BMI) was 23.8±3.5 (kg/m^2^). “Mother-to-child” (n=79, 56.4%) was the most frequent relationship. More than 90% of our participants were volunteers to donate again and recommend LDLT to somebody else; to be exact, 91.8% and 91.7%, respectively (Table 2).

**Table 2:** Donors characteristics. Values are either mean±SD or n (%)

Variables		Statistics
Age, yrs		32.1±7.3
Body mass index, kg/m^2^		23.8±3.5
Sex	Male	57 (40.7)
	Female	83 (59.3)
Marital status	Married	134 (95.7)
	Others	6 (4.3)
Education	Illiterate	4 (2.9)
	Primary school	27 (19.3)
	Secondary school	28 (20.0)
	High school	36 (25.7)
	Diploma and higher	45 (32.2)
Employment	Employed	61 (43.6)
	Unemployed	74 (52.9)
	Pensioned	5 (3.5)
Ethnicity	Fars	92 (65.7)
	Arab	14 (10.0)
	Turk	12 (8.6)
	Kurd	10 (7.1)
	Lor	4 (2.9)
	Balooch	1 (0.7)
	Others	7 (5.0)
Relationship to recipient	Mother	79 (56.4)
	Father	49 (35.0)
	First relative(grand-uncle-aunt)	5 (3.6)
	Daughter	4 (2.9)
	Spouse	1 (0.7)
	Others(second degree relatives)	2 (1.4)
First learned about LDLT	Transplant team	98 (70.5)
	General physician	18 (12.9)
	Family	15 (10.8)
	Others	8 (5.7)
Source of income	Sick leave	11 (7.9)
	Savings	56 (40.0)
	Others	73 (52.1)

Operative Details and Outcomes

Among 140 living liver donors, 75 (72.0%) underwent left lateral; 24 (23.1%), left lobe; and 5 (4.8%), right hepatectomy. The mean±SD of operative time was 233.8±46.9 min. The mean±SD of hospital stay and ICU stay were 4.4±2.3 and 2.8±0.9 days, respectively. There was no death among our donors. Twenty-two (15.7%) complications were recorded among our participants. The intra-operative data of all the donors were collected retrospectively (Table 3).

**Table 3:** Intra- and post-operative characteristics of studied donors. Values are either mean±SD or n (%).

Characteristic		Statistics
Mode of donor hepatectomy	Left lateral	75 (72.0)
	Left lobe	24 (23.1)
	Right lobe	5 (4.8)
Pre-operative biochemical profile	Total bilirubin, µmol/L	0.9±0.5
	Alkaline phosphatase, IU/L	183.9±61.9
	Hemoglobin, g/dL	14.0±1.8
Post-operative biochemical profile	Total bilirubin, µmol/L	1.7±0.9
	Alkaline phosphatase, IU/L	152.7±50.4
	Hemoglobin, g/dL	12.2±1.7
	Operation time, min	233.8±46.9
	ICU stay, day	2.8±0.9
	Mean hospital stay, day	4.4±2.3
	Mean complete recovery time, month	3±1.4
	Post-operative complications according to Clavien system	22 (15.7)
Grade 1(n=7, 31.81%)	Fever of unknown origin	1 (5)
	Atelectasis	3 (13.6)
	Neuropraxia	1 (5)
	Mild pleural effusion treated conservatively	2 (9)
Grade 2 (n=7, 31.81%)	Wound infection requiring antibiotics	6 (27.3)
	Blood transfusion	1 (5)
Grade 3A (n=4, 18.18%)	Intra-abdominal abscess	1 (5)
	Bile stricture	1 (5)
	Bile leakage treated with percutaneous drainage, ERCP	2 (9)
Grade 3B(n=4, 18.18%)	Intra-abdominal bleeding requiring laparotomy	4 (18.2)
Grade 4A		0 (0)
Grade 4B		0 (0)
Grade 5		0 (0)

SF-36 Results

The mean±SD of PCS and MCS scores were 48.8±14.6 and 50.1±6.9, respectively (Table 4). Improvement was reported by 131 (93.6%) recipients; 9 (6.4%) died. There was significant (p=0.011) relationship between the recipient outcome and donors’ PCS. No significant (p=0.449) relationship was observed between the recipient outcome and donors’ MCS score. There was no significant correlation between age and the quality of life scales measured.

**Table 4:** Comparison of mean±SD of SF-36 scores between living liver donors and general population of Iran

Scales	Our study (n=140)	Ali Montazeri, *et al* (n=4163)	p value
PF	70.4±19.1	85.3±20.8	<0.001
RP	73.0±48.3	70.0±38.0	0.363
BP	78.5±23.1	79.4±25.1	0.675
GH	53.1±12.9	67.5±20.4	<0.001
VT	45.8±10.6	65.8±17.3	<0.001
SF	79.0±19.6	76.0±24.4	0.152
RE	76.6±49.7	65.5±41.4	0.002
MH	43.9±9.3	67.0±18.0	<0.001
PCS	48.8±14.6	—	—
MCS	50.1±6.9	—	—

Males had a significantly higher scores in physical functioning scale compared with females. However, there was no significant difference in other scales between males and females. No significant difference was found in quality of life scales between employed and non-employed donors (Table 5).

**Table 5:** The Effect of sex, education, and employment on the mean±SD SF-36 domains

Scales	Education			p value	Sex		p value	Employed		p value
	Intermediate and lower (n=59)	High school and diploma (n=54)	College (n=27)		Male (n=58)	Female (n=82)		Yes (n=66)	No (n=74)	
PF	69.9±18.7	69.6±19.0	73.4±20.8	0.678	74.3±18.3	67.7±19.3	0.046	73.5±19.7	67.8±18.4	0.084
RP	72.5±51.1	75.9±52.5	68.2±30.4	0.801	76.2±40.3	70.7±53.4	0.505	69.9±28.9	75.6±60.0	0.486
BP	78.4±24.8	76.8±22.1	82.2±21.8	0.634	79.5±21.4	77.8±24.4	0.684	80.6±21.2	76.8±24.7	0.329
GH	53.5±12.1	52.7±10.2	53.2±18.8	0.948	54.4±13.8	52.5±12.2	0.462	54.1±14.8	52.3±11.0	0.420
VT	47.0±10.9	44.4±10.8	45.9±9.4	0.426	46.0±9.9	45.7±11.1	0.869	45.7±10.5	45.9±10.7	0.876
SF	78.4±19.2	79.7±18.0	78.8±24.1	0.945	80.1±20.5	78.2±19.1	0.561	79.4±20.8	78.6±18.7	0.795
RE	73.7±53.4	81.1±52.9	74.3±31.7	0.711	79.3±42.7	74.7±54.3	0.599	73.9±32.2	78.9±60.8	0.556
MH	44.0±9.7	43.5±9.1	44.4±8.9	0.912	43.9±8.94	43.9±9.6	0.990	43.8±9.4	44.0±9.2	0.937
PCS	48.2±14.4	48.9±14.8	50.2±15.0	0.937	51.2±13.8	47.2±14.9	0.109	50.1±14.0	47.8±15.0	0.349
MCS	50.1±6.7	49.6±7.5	50.8±6.3	0.794	50.4±6.65	49.8±7.1	0.590	50.1±6.6	50.0±7.2	0.892

## DISCUSSION

One of main issues in liver transplantation is shortage of deceased donors. Using living donors is one of the solutions for this problem. However, one of the main items in this field is the health of donors [13, 14]. We studied the safety of living donor liver transplantation retrospectively based on different factors in the current study. A total of 15.7% of our participants experienced a complication; no death was reported. 

Studies from different countries report various results. Post-operative complications occur in 28% of living liver donors in Brazil. The rate was 13.2 in Japan and 11.6% in Pakistan. No mortality was reported [15-17]. The reported wound infection and biliary complication rates were the same (5.9%) in Japan. The most common post-donation complications were bile leak, incisional hernia, pneumonia, and intra-abdominal collection in Pakistan [16, 17]. A systematic review on safety of living donors reported a donor mortality rate of 0.2%, and a median donor morbidity rate of 16% (range: 0%–100%). Biliary complications and infections were the most frequent complications [18]. A study from Japan on 28 donors showed that wound-related physical symptoms (24%) and anxiety (19%) were the common reported complications [19]. A study from South Asia reported an overall morbidity rate of 23%; wound infections (4.3%) was the most common complication [20]. Wound infection was the most common complication among our participants too. Using laparoscopic hepatectomy may reduce wound-related symptoms. 

A study conducted in the US reported that of 740 LDLT (707 right lobes), 39% developed at least one complication in the first year [5]. Majority of donors in the US underwent right lobe hepatectomy that could explain the difference observed between their complication rate and ours.

The most complications of our studied donors were classified as Clavien grades 1 and 2, which was consistent with other studies [5, 6]. We found mean±SD scores of 48.8±14.6 for PCS and 50.1±6.9 for MCS. Post-operative donors PCS and MCS scores were almost near to the average of the general population in Iran [12, 21]. There were significant differences between domains of physical functioning, general health perceptions, vitality, emotional role functioning, general mental health obtained among donors in our study (Table 4) and those reported in general population in Iran [22]. 

Mental health and vitality scores were below the average in our study (Table 4). A study conducted in general population in Iran showed that mental health-related quality of life was lower than the physical health-related quality of life [12]. It could be due to the anxiety and depression before and after donation. On the other hand, the majority of donors were parents of recipients, which could explain this finding.

A review article reported that physical scores of quality of life decrease in the first three months after donation; they return back to the baseline within six months in the majority of donors. Mental scores are unaffected during the donation process [8, 19]. The results we obtained for PCS and MCS domains were almost near to those values reported three months after donation in another study [23]. The result of two cohort studies show that surgery-related complications do not significantly change the quality of life by itself in the majority of donors [3, 23]. 

Donation experience was positive among our participants. The majority (91.8%) of our donors volunteered to donate again. Living liver donors at the University of Minnesota shared the same sentiment [24]. 

A study shows that the majority of donors have a recovery time of one year [5]. Approximately one-third of our donors had post-donation follow-up less than one year at the time of study. This is a limitation of our study. We should have included donors with more than one-year post-donation follow-up too. Our study had other limitations. It was a cross-sectional study and thus we could not determine pre-donation SF-36 scores. Although all medical records were studied in detail, the accuracy of the results is not comparable with longitudinal studies. Given that the studied donors were from healthy population, it would have been better to design a longitudinal study and evaluate the quality of life of the donors before and after the donation. 

In conclusion, most living donors sustain a quality of life near average of the general population after donation. This means that living donation does not negatively affect the quality of life of donors in Iran.
